# Book Notices

**Published:** 1860-04

**Authors:** 


### BOOK AND PAMPHLET NOTICES.

A MEDICO-LEGAL TREATISE ON MALPRACTICE AND MEDICAL EVIDENCE; COMPRISING THE ELEMENTS OF MEDICAL JURISPRUDENCE. By JOHN ELWELL, M. D., Member of the Cleveland Bar. New York and Cleveland: Alfred Elwell & Co., 1860. Pp. 588. From the publishers.

This book appears to be a full and perfect treatise on the subjects of which it professes to treat.

In how far it is correct in the rules which it lays down, we are unable, from a personal examination, to say. It has, however, received the endorsement of some of the most eminent legal men, as well as of physicians, in this country, and from a cursory examination it appears to be full, accurate, and prepared with care.

Such a book should be in the hands of physicians generally, who are too often not aware of the nature of the responsibility which they assume in accepting the charge of surgical cases.

A PRACTICAL TREATISE ON OPERATIVE DENTISTRY. By J. TAFT, Professor of Operative Dentistry, in the Ohio College of Dental Surgery. With eighty illustrations. Philadelphia: Lindsay & Blakistone. Chicago: Jones & White, Dental Depot.

The author of this book seems less anxious to be regarded as the originator of the theories he adopts, and the practice he recommends, than to give a well-digested and systematized *resume* of the theories of dental diseases, as adopted by the best Dental Pathologists, and the practice prevailing among the better class of dentist in treating these diseases.

In a brief but comprehensive "Introductory," he describes the varies kinds of *tartar* that deposits upon the teeth, and the best method of removing it; the nature, cause, and effect of atrophy, exostosis, denuding of the enamel, chemical abrasion, necrosis of the teeth, &c., &c.

He then takes up and describes very minutely, the characteristics and causes of "caries of the teeth."

The causes are considered under two general divisions, Predisposing and Exciting. Among the former are hereditary defects—functional derangements, which prevent a proper elimination or appropriation of materials necessary for the perfect structure of the tooth—vitiated secretions of the mouth—badly performed dental operations, and the like.

Among the most active of the exciting causes, all decomposition of animal and vegetable matter between and around the teeth—contact of hydrochloric acid with the teeth, consequent upon the eructation of the fluids of the stomach of dyspeptics—medical preparations, and condiments containing acids—acids formed by the decomposition of substances in the mouth, in consequence of a constant galvanic action going on between different metals used in filling natural teeth and plates for artificial teeth. The author then passes on rapidly, making some valuable suggestions in regard to the treatment of superficial or slight caries of the teeth, and comes to the important subject of *filling teeth.*

In discussing the merits and demerits of the various materials used for this purpose, gold and tin are considered to be the only ones of much real value. Amalgams or cements, which must always contain mercury, and are usually made by mixing mercury, tin, and silver, together, are very properly regarded as "*vile compounds*, never to be used as fillings for the teeth.

Then follow descriptions and cuts of instruments necessary and proper for preparing cavities and introducing fillings—forming of cavities, and the proper method of introducing and finishing fillings—treatment of exposed nerves—setting pivot teeth—necessary instruments for, and the method of using them in extracting the various classes of teeth and roots—treatment of undue hemorrhage after the extraction of teeth, etc., etc.

It will be seen from this somewhat detailed, though quite imperfect summary of contents, that this work is just what its title indicates. "*A Practical Treatise on Operative Dentistry.*"

Dr. Taft evinces an intimate and practical knowledge of what he is writing about, and in all cases uses such simple and well chosen language that there is no mistaking the idea he wishes to convey—making this one of the most valuable books ever published for the use of students of Dental Surgery. A.

---

I. THE DIAGNOSIS, PATHOLOGY, AND TREATMENT OF DISEASES OF THE CHEST. By W. W. GERHARD, M. D., one of the Physicians to the Pennsylvania Hospital, etc. 4th edition, revised and enlarged. Philadelphia: Lippincott & Co.; 1860. Pp. 448. From S. C. Griggs & Co.

II. A PRACTICAL TREATISE ON THE DIAGNOSIS, PATHOLOGY AND TREATMENT OF DISEASES OF THE HEART. By AUSTIN FLINT, M. D., Professor of Clinical Medicine, etc., in the New Orleans School of Medicine, etc. Philadelphia: Blanchard & Lea, 1859. Pp. 473. From Keen & Lee.

The work of Dr. Gerhard has been long before the public, and its character is now well established.

The present edition, however, contains about one hundred pages of new matter, and many additions relating to Pneumonia, Pulmonary Consumption, and Diseases of the Heart. The work is concise, accurate, and well adapted to supply the wants of the student and the practitioner. We extract passages referring to the use of cod liver oil and the phosphates, which are of particular interest:

"The following may be given as the results of the use of cod liver oil in the medical wards of Pennsylvania Hospital during the last six months, (1849–50:)

1st. *Of the Oil.*—That the light-colored oil can be taken without difficulty by patients whose stomachs have steadily rejected the brown oil.

2d. *Of the mode of administration.*—That a few of the patients have taken the oil without any adjunct to disguise its taste. That its nauseating properties are corrected by its administration with milk; but that its taste is most effectually disguised by the froth of porter.

3d. *Of the time of its administration.*—As a general rule it has been taken before meals, but in four instances where it was not tolerated before meals it was readily taken after meals.

4th. *Of diarrhœa as a contra-indication to its use.*—The existence of diarrhœa is not a positive contra-indication to its use. In three instances in which patients were thus affected, no increase of the symptoms was produced by its use, and no diminution by its abandonment. In a fourth instance, when the diarrhœa had previously existed, the discharge appeared to be increased by the exhibition of the oil, and abated with its withdrawal.

5th. *Of its effects in cases of phthisis pulmonalis.*—Patients using the oil have increased in flesh, in weight, and strength. While using the oil, their cough and expectoration have diminished; that with some, hectic and rigors have entirely disappeared. Six of them have been so much benefited as to leave the hospital and resume their former occupations. In one instance, a patient who entered the hospital with cough, copious purulent expectoration, extreme emaciation, inability to leave his bed, and with the physical signs of a cavity under the left clavicle, after six months use of the oil, left the hospital weighing 140 pounds, with little or no cough, no hectic or rigors, and with an almost entire absence of expectoration, the physical signs having greatly diminished.

6th. *Of the physical signs.*—The improvement of the physical signs is not coincident with that of the general symptoms.

7th. *Of its use in general scrofula.*—In scrofulous diseases where there was no reason to suspect the existence of pulmonary tubercles, the improvement of the patient's health has been very decided.

8th. *Of congestion of lungs as produced by cod liver oil.*—There has been no decided evidence of such a result following the use of the oil in the preceding cases. [Two patients of the twenty, while using the oil, had severe attacks of hæmoptysis, but there was no reason to refer them to the use of the remedy.]

9th. In those cases which have terminated fatally, the appetite, the nutrition and strength of the patient appeared for a time to be decidedly increased; the life of the patient seemed to be in this manner, temporarily protracted; but for a few weeks immediately preceding death the remedy seemed to have entirely lost its value.

10th. *Length of time, etc.*—To be of any decided permanent benefit, its use must be steadily persisted in. It should be continued even after the most striking symptoms of the disease have, in a great measure disappeared.

Another mode of treating phithisis which has attracted much notice within the last few years is the use of different phosphatic salts, given in connection with one another. These are the phosphates of iron, soda, lime, and potash. This plan was proposed by Dr. Churchill, and was founded on the supposition that the addition of these salts to the blood would enable it to withstand the tendency to tuberculous deposit. I have been chiefly in the habit of giving these salts simply mixed with water, directing the patient to shake it up before taking it, so as to suspend the phosphate of iron, which is of course insoluble. The mixture is made according to the following formula:

℞.—Sodæ phosphat, ʒ iv; Calsis phosphat, ʒ ij; Potassæ phosphat, ʒ i; Ferri phosphat, ℈ ij; Aq. Rosæ, ℥ vi.—M.

S. A teaspoonful after breakfast and dinner, first shaking the bottle. If the substance is well shaken, the phosphate of iron may be of course sufficiently suspended to be taken in a proper dose.

Mucilage should not be added, as it would soon spoil. My friend Dr. Jackson has been is the habit of giving the same medicine in sirup, making a more uniform and prettier preparation, but one which I do not like so well as a simple suspension of these salts. If, however, this preparation should be preferred, the best mode of making it is one given by Mr. Parrish in the Am. Joun. of Pharmacy for 1857, vol. xxix. p. 572.

The use of these phosphates became very general in Philadelphia, but has much diminished within the past year or two. This comparative disuse of them has arisen simply from the doubt that remained in the minds of those who employed them as to their utility."

The volume of Dr. Flint is new. Its scope and object are sufficiently shown by the following extract from the preface:

"As regards the order in which the different diseases are considered, the plan usually pursued may be said to be synthetical, inflammatory affections being taken up first, and afterward the lesions which are, to a considerable extent, the results of inflammation. A method which may be distinguished as analytical, has appeared to the author preferable. Pursuing this method, the work commences with the consideration of organic affections. Enlargement of the heart, occurring often consecutively to other lesions, takes precedence. To this subject the first chapter is devoted. Lesions affecting the walls of the heart naturally come next in order. These constitute the subject of the second chapter. Valvular lesions are then considered, occupying two chapters, and a chapter is devoted to congenital malformations. Several affections which are incidental to diseases of the heart, are treated of in a distinct chapter. Then follow the inflammatory affections, and, afterward, functional disorders of the heart, three chapters being allotted to these classes of disease. Finally, thoracic aneurisms, which claim considerations in connection with diseases of the heart, are made the subject of the concluding chapter."

In regard to the merits of the work we have no hesitation in pronouncing it full, accurate, and judicious. Considering the present state of science, such a work was much needed. It should be in the hands of every practitioner.

Without intending to enter into the discussion of any points concerning which a difference of opinion might exist, we extract our author's views on the hygienic and medical treatment of certain valvular lesions, which we deem of importance.

"Undue excitement and overtasking of the heart induce weakness and favor dilatation. The muscular power here, as in other situations, is exhausted by too great exertion, and the walls yield more readily to distension, under these circumstances, from the accumulation of blood within the cavities. The causes, already referred to, which excite unduly and overtask the heart, viz: excessive muscular exercise, mental excitement, the intemperate use of alcoholic stimulants, etc., are, therefore, to be avoided with respect to the second, not less than the first object of treatment. Exercise, however, within certain limits is, highly important with a view to the preservation of the power of the heart's action. Patients affected with obstructive or regurgitant lesions, will retain a compensatory vigor of the heart, and the epoch when dilatation succeeds hypertrophy will be postponed for a longer period, by habits which involve a judicious amount of exercise, than by a life of complete repose. Active occupations, whether pursued as a calling or for amusement, or with reference merely to exercise, should not be abandoned. Persons under the necessity of performing daily manual labor will do better to continue to work, so far as they are able without inconvenience, than to become fixtures in the wards of a hospital. They who are above this necessity should either follow some active pursuits, or engage in sports which demand a certain amount of physical activity. Indolence or inaction of the muscular system tends to produce weakness of the heart and favors fatty degeneration, thereby contributing to the production of dilatation rather than hypertrophy. The rules which should govern exercise have already been considered in connection with the treatment of hypertrophy, to which the reader is referred.* These rules are applicable to cases of valvular lesions, with or without hypertrophy.

* Chap. I, page 72.

They are, of course, not to the same extent applicable to cases in which the lesions have already led to dilatation.

The diet suited to obviate a tendency to weakness and dilatation is that best adapted to healthy nutrition. Healthy nutrition, and thereby the muscular vigor of the heart, require blood rich in nutritive materials. A poor and insufficient diet tends to hasten the evils resulting from valvular lesions. The diet should embrace a fair proportion of animal food. Liquids should be taken sparingly, the object being to secure a good quality, but not to increase the quantity of blood. Restrictions, as respects fatty substances and those readily converted into fat, are important, if there are grounds to suspect a disposition to fatty degeneration. The articles of food should be adapted to the digestive powers. The action of the heart, as is well known, is liable to be disturbed through its sympathetic connection with the stomach, when digestion is labored or imperfect. Dyspeptic disorders will claim appropriate treatment. Tonics and stimulants, in moderate quantity, are indicated whenever the digestive powers are enfeebled. Exercise in the open air, within proper limits, is important with reference to its influence on digestion. Cheerfulness and mental recreation are desirable for the same end.

Opposite conditions of the blood alike tend to weakness and dilatation, viz: plethora and anæmia. If the blood be too abundant, and the red globules in excess, the heart is overtasked and unduly stimulated. Bloodletting, under these circumstances, may be appropriate. But it should be employed with discrimination and great circumspection, inasmuch as the impoverishment caused by its injudicious employment is a condition worse than plethora. In general, other methods of depletion, which are not spoliative, are to be preferred, viz: saline laxatives and diuretics, in conjunction with a dry diet. Anæmia is a far more unfavorable condition than plethora, and claims efficient treatment with chalybeate tonics, nutritious diet, etc. The symptoms referable to the heart, in some cases of valvular lesions, are, in a great measure, due to functional disorder incident to anæmia, and when the anæmic condition is removed, all the symptoms may disappear. This fact should be borne in mind. The practitioner is liable to consider all the symptoms as resulting directly and exclusively from the lesions, and, consequently, is led to exaggerate the immediate danger from the latter. Patients who suffer much from palpitation, etc., when anæmia is conjoined with valvular lesions, may experience no inconvenience when the blood is restored to its normal condition. This is intelligible in view of the well-known fact that anæmia often gives rise to functional disorder of the heart when this organ is free from organic disease."

